# A New MANET Wormhole Detection Algorithm Based on Traversal Time and Hop Count Analysis

**DOI:** 10.3390/s111211122

**Published:** 2011-11-28

**Authors:** Jonny Karlsson, Laurence S. Dooley, Göran Pulkkis

**Affiliations:** 1 Department of Communication and Systems, The Open University, Walton Hall, Milton Keynes, MK7 6AA, UK; E-Mail: l.s.dooley@open.ac.uk; 2 Department of Business, Information Technology and Media, Arcada University of Applied Sciences, Jan-Magnus Janssons plats 1, Helsinki 00550, Finland; E-Mail: goran.pulkkis@arcada.fi

**Keywords:** mobile networks, MANET, MANET security, routing security, wormhole attack, hop count, traversal time, WAP, DelPHI, MHA

## Abstract

As demand increases for ubiquitous network facilities, infrastructure-less and self-configuring systems like Mobile *Ad hoc* Networks (MANET) are gaining popularity. MANET routing security however, is one of the most significant challenges to wide scale adoption, with wormhole attacks being an especially severe MANET routing threat. This is because wormholes are able to disrupt a major component of network traffic, while concomitantly being extremely difficult to detect. This paper introduces a new wormhole detection paradigm based upon Traversal Time and Hop Count Analysis (TTHCA), which in comparison to existing algorithms, consistently affords superior detection performance, allied with low false positive rates for all wormhole variants. Simulation results confirm that the TTHCA model exhibits robust wormhole route detection in various network scenarios, while incurring only a small network overhead. This feature makes TTHCA an attractive choice for MANET environments which generally comprise devices, such as wireless sensors, which possess a limited processing capability.

## 1. Introduction

Mobile *Ad hoc* Networks (MANET) are self-configuring arrangements of small portable devices interconnected by wireless links, with no fixed infrastructure like base stations and dedicated routers. They can be deployed in a diverse range of application domains including wireless sensor and vehicular networks, military communications, and as a viable solution for Internet connectivity in *fourth-generation* (4G) networks, especially where nodes are located out of radio range, as for example in underground transport systems.

Given their inherent self-configuring nature, each MANET node participates in the routing process, in addition to its other activities. A number of dedicated MANET routing protocols have been proposed, with the reactive protocols *Ad hoc* On-Demand Distance Vector (AODV) [1] and Dynamic Source Routing (DSR) [2], being the most widely adopted. Security mechanisms have not always been an integral feature or priority in routing protocol development, with the underlying assumption being that all MANET nodes were trustworthy. Security extensions to MANET routing protocols have subsequently evolved, such as Secure AODV (SAODV) [3] and ARIDANE [4], though the corollary of not having trusted routing means MANET routers are especially vulnerable to malicious node activity leading to potentially severe disruption in network communications. Such disruption can range from deliberately ignoring the routing protocol through to tampering with routing packets. For example, to save energy a selfish node may simply not take part in the routing process leading to packet loss, while a malicious node can launch serious network attacks such as, rerouting packets from their original path to an erroneous destination node and even to stealing the identity of a node.

One of the most serious MANET security threats is the wormhole attack [5] which is characteristically launched by two malicious nodes, with one capturing routing packets at a particular node location and then tunnelling them through to the other malicious node located some distance away, which in turn relays tunnelled packets to its neighbouring nodes. As a consequence, two nodes from distant parts of the network appear much closer to each other than they are in reality. Once the wormhole has been successfully established, the malicious nodes can disrupt network operation by dropping packets, or launching more pernicious attacks such as eavesdropping and packet sniffing. It has been theoretically proven that the strategic placement of a single wormhole in a uniformly-distributed MANET can either disrupt or control nearly a third of all network communications [6].

There are two classes of wormhole attack: Hidden Mode (HM) and Participation Mode (PM) [7]. HM wormhole nodes are invisible from legitimate nodes as they do not process routing packets. They simply capture, tunnel and forward packets to each other and never appear in routing tables. In contrast, PM wormhole nodes are visible during the routing process since they process routing packets as any normal node. Aside from relaying routing packets to its neighbours, a PM wormhole node tunnels routing packets to the other PM node, giving it the opportunity to deleteriously control network performance.

A shortcut link between either two HM or PM wormhole nodes can be established by either an In-Band (I-B) or Out-of-Band (O-B) channel [8]. The former is defined when the wormhole nodes tunnel packets to each other through legitimate nodes in the network, while the O-B channel connects two malicious nodes via an external communication link such as a network cable or directional antenna.

From a MANET perspective, wormholes are especially difficult to detect for two reasons:

- There is the latent variability in the environment in terms of the number of users, their locations and the applications they are running. A MANET can operate either as a closed network, where a legitimate node can be easily separated from unauthorised nodes, or as a highly dynamic network, exhibiting considerable intermittent nodal connectivity so making it very challenging to distinguish malicious from legitimate nodes. Furthermore, network devices can vary from small energy-constrained computing devices with limited hardware capability though to powerful personal computers.
- There is the diversity of feasible wormhole attacks, *i.e.*, PM, HM, I-B and O-B channels. Each wormhole type has its distinct characteristic providing the opportunity to launch attacks in many different modes, with each mode imposing its own set of challenges for any detection mechanism. In addition, cognisance of the incidences of erroneous wormhole identification, so called False Positive (FP) must be considered in any proposed detection paradigm.

These reasons provided the motivation to investigate a generic wormhole detection methodology with the aim of achieving both consistently high detection rates under a range of network conditions, and having the capability to manage all wormhole variants. Before introducing the new detection algorithm, a review of some of the existing prevention and detection strategies for MANET wormholes is presented.

### 1.1. Research Background

Wormhole detection mechanisms can broadly be divided in two categories: neighbour validation and end-to-end detection. Examples of the former include packet leashes [5] and [6] which involve appending information to a packet relating to either distance or time, to limit that packet's admissible transmission distance. This is an effective preventative strategy against HM wormhole attacks because outlying legitimate nodes appear as neighbours and a packet can be dropped if the transmission distance/time of a received packet is unrealistically long. Such countermeasures however, are unable to detect PM wormholes because malicious nodes appear in the routes and are considered as valid neighbours.

In contrast, end-to-end detection mechanisms typically measure and analyse network node and route features like the frequency of node appearances in routes, the geographical positions of nodes, the Round Trip Time (RTT) performance of data packets, and Hop Counts (HC) across multiple routes. Many proposals adopt cryptographic techniques to protect the routing packets such as in the AODV and DSR extensions, SAODV and ARIDANE. Such packet security however, can only be effective against the threat of PM wormholes, and not HM wormholes because these do not need to process any routing packets. Moreover, cryptography is only applicable within closed MANET environments comprising trusted nodes, such as a company intranet where network access can be restricted to employees. In an open dynamic MANET with nodes continuously joining and leaving the network, a reliable check for trustworthiness whenever a node joins the network is not feasible. For instance, in an underground transport system MANET where the network structure is dynamic and the number of users is high, it would be impossible to distinguish a malicious user from a legitimate user.

Strategies that measure and analyse the frequency of node appearances in routes [9–11] have been an effective countermeasure for both PM I-B and O-B wormhole nodes because they appear more often in routes. If the MANET is highly dynamic however, it can be difficult to maintain the frequency data unless either a centralized node is assigned the task of storing the data or it is continually shared with other MANET nodes. The ensuing solution can thus impose a high load upon the network bandwidth. Furthermore, HM wormholes cannot be detected using this approach as they are invisible and do not appear in any route.

Geographical location based solutions including First End-to-End Protocol to secure *ad hoc* networks with Variable Ranges (FEEPVR) [12] and Simple and Efficient End-to-End Protocol (SEEEP) [13], utilise information concerning node positions to achieve detection of both I-B and O-B channel wormholes launched in either HM or PM. The main drawback of these techniques is that they require every node to have a positioning device, *i.e.*, a Global Positioning System (GPS) facility which pragmatically precludes adoption in MANET applications, where nodes tend to be small low-cost computing devices like sensors.

In contrast, RTT-based solutions including Delay Per Hop Indication (DelPHI) [14], Wormhole Attack Prevention (WAP) [15], and Transmission Time-based Mechanism (TTM) [16] do not require dedicated positioning hardware and are based on the premise that a route with a small HC will concomitantly have a small RTT measure. If the RTT per HC of a specific route is higher than a pre-calculated threshold, the route is considered to be a wormhole route. RTT-based solutions are effective in detecting both HM and PM I-B wormhole types, provided that variations in packet processing delay times on the nodes are small. In practical MANET scenarios however, this assumption will not always hold because of the unpredictability of network traffic loads, which significantly compromises the reliability of RTT solutions. In addition, RTT methods cannot detect either HM or PM O-B wormholes because the time delay on these wormhole links is negligibly small compared to the variation in packet processing delays on intermediate nodes.

An alternative HC-based technique that does not require accurate RTT measurement is Multi Hop-count Analysis (MHA) [17]. This provides an efficient solution from both a computational and hardware perspective, but is only effective under specific network conditions. MHA modifies the AODV route discovery protocol to identify several unique routes between the source and destination nodes. A route with a markedly lower HC than other routes is then assumed to include a wormhole and is avoided in network communications. MHA can detect both PM I-B and O-B wormholes, but its key assertion is that a wormhole route always exhibits a significantly lower HC than other routes. If there is no HC disparity between routes, which is often the case in real-world MANET scenarios, the detection rate falls and a correspondingly higher FP number occurs. MHA also targets a fixed number of unique paths during route discovery by using a graylist to distinguish already used nodes. This compromises HM wormhole detection because HM nodes do not appear in the graylist, so there is the inherent risk that all identified routes in fact, traverse a HM wormhole.

A recurring feature of many existing wormhole detection techniques is that to some degree, they incur a price in terms of their wormhole coverage capability, computational complexity, route analysis and time measurements, hardware requirements and network outlay to achieve improved detection rates. This ultimately militates against their applicability in dynamic MANET environments, where nodes normally have limited processing and power capability. This provided the impetus to investigate a new generic detection strategy to provide enhanced performance for all wormhole variants, whilst affording a lightweight implementation from a computational and network overhead perspective.

This paper presents a new packet-based Traversal Time and Hop Count Analysis (TTHCA) wormhole detection algorithm designed as an extension to the AODV routing protocol combining some of the latent benefits of RTT-based techniques with HC analysis, to afford superior detection performance and full wormhole type coverage under a variety of network conditions. Using link delay analysis to detect a wormhole infected route, TTHCA crucially excludes packet processing delays from the RTT measurements on each intermediate node, to provide a Packet Traversal Time (PTT) measure. A wormhole route is identified if the PTT in relation to the HC of a route is unrealistically high. TTHCA significantly improves both wormhole detection performance and FP occurrence, compared to RTT-based mechanisms, since variations in the propagation speed of a data packet are negligible which means the traversal time of a data packet is proportional to the distance it has travelled.

In HC analysis terms, TTHCA adopts the graylist concept [17] for route discovery to prevent wormhole nodes from appearing in multiple routes, whilst embedding greater flexibility in two important aspects. Firstly, it is able to identify wormholes which do not always have the lowest HC route, and secondly, it enables HM wormholes to be effectively detected. TTHCA is not dependent on a fixed number of route samples, but instead the route discovery process is repeated until a safe route is found. TTHCA concomitantly provides low network overheads as it does not have recourse to broadcast routing messages more than once in wormhole-free networks.

The rest of the paper is organised as follows: the new TTHCA algorithm is presented in Section 2, while Section 3 analyses both its wormhole detection performance and FP occurrence rate in comparison with existing techniques, together with a statistical significance test evaluation. Finally, some conclusions and future work are discussed in Section 4.

## 2. Packet-Based Traversal Time Hop Count Analysis (TTHCA)

The rationale behind TTHCA was to develop a wormhole detection algorithm able to manage all variants of wormholes and implementable for all types of MANET devices and network scenarios, without incurring significant computational and network costs. TTHCA has been designed as an extension to the AODV routing protocol allowing the source node to receive, in addition to HC information, the total routing packet processing delay *ΔT_TOT_* during an AODV route discovery phase. Using *ΔT_TOT_* together with the RTT of a route, the source node can calculate the PTT of the route and compare it with the HC to identify potential wormhole routes. If PTT in relation to HC is greater than a pre-set threshold, the route discovery procedure is repeated until a safe route is found. To obtain *ΔT_TOT_* for a route, each intermediate node must measure the packet processing times of both the RREQ (*ΔT_RREQ_*) and RREP messages (*ΔT_RREP_*) and include these values in a new parameter within the RREP packet.

As alluded in Section 1.1, TTHCA seeks to integrate the core concepts of RTT-based measurement with HC analysis, and while these separate mechanisms have proven effectual as wormhole detection techniques, their underlying design imposes certain inherent limitations in both achievable detection rates for MANET scenarios and the wormhole types each approach can identify. Before presenting the new TTHCA algorithm in detail, the next section briefly explores some of these constraints which TTHCA was specifically designed to address.

### 2.1. Limitations and Benefits of Current RTT and HC-Based Wormhole Detection Algorithms

RTT-based techniques such as WAP and DelPHI, are based on the supposition that the RTT of a route is closely related to its distance and HC, which is true provided all network nodes exhibit a near constant packet processing delay. WAP uses a Wormhole Prevention Timer (WPT) threshold for the maximum permitted RTT per HC; with the underlying assumption that the processing times of routing packets on intermediate nodes are negligibly small. In contrast, DelPHI assumes that a wormhole route has a significantly higher RTT than a normal route. In practical MANET environments however, packet processing delays are high relative to the traversal times and exhibit a high variance. There exists the probability that a healthy route with a short distance and low HC will produce a high RTT due to a momentary high packet processing delay at one of the intermediate nodes caused for instance, by traffic congestion. Conversely, a wormhole infected route with a long distance may provide a low RTT due to low packet processing delays on all intermediate nodes. For these reasons, wormhole detection performance is compromised and FP occurrences increased when RTT-based solutions are used in realistic MANET environments, as will be evidenced in Section 3.

**Figure 1.**
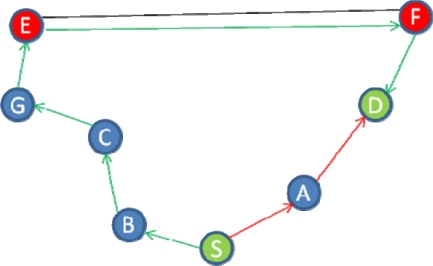
MANET example where MHA fails to detect a PM O-B wormhole.

The HC analysis scheme in MHA is predicated on identifying routes with a significantly lower HC than other routes, and then classifying these routes as wormholes. This is a successful detection strategy provided that the wormhole route always has the lowest HC. In MANET scenarios where this assumption is not valid, the corresponding detection performance is compromised. The example in Figure 1 illustrates the problem, where S and D are the source and destination nodes respectively, while E and F are two malicious nodes forming a PM O-B wormhole.

The shortest route in this example is two hops (S-A-D), while the alternative route is six hops (B-C-G-E-F-D), so MHA prevents a packet being routed via the shorter route because it has a significantly lower HC. As a consequence, data packets will be routed through the wormhole.

The graylist attribute used in MHA for route discovery is an effective way of finding multiple unique routes between a source and a destination node since it ensures that PM wormhole nodes for instance, cannot appear in more than one route per discovery phase. This means two different routes are sufficient to be able to detect one PM wormhole route based on HC analysis. The number of requested routes per route discovery in MHA is fixed and known as the Route Reply limit (*RREP_lim_*). HM wormhole nodes cannot be added to the graylist since they do not appear in routing tables, so there is a risk that all requested routes traverse a wormhole link. This risk can be reduced by increasing *RREP_lim_*, though this increases the route discovery delay overhead which is undesirable. In the results analysis (Section 3), it will be shown that when MHA is applied to realistic MANET environments with randomly distributed nodes, the wormhole detection rate is compromised, with a commensurate increase in the occurrence of FP wormholes. It also reveals that the existence of HM wormholes further degrades the detection performance of MHA.

Despite these drawbacks MHA, WAP and DelPHI exhibit qualities that can be exploited in a unified wormhole attack detection framework. They offer for example, a low overhead solution in terms of hardware, computation and throughput. These features provided the motivation to investigate how they could be combined and extended to improve detection rates, FP occurrence performance and crucially, broaden the detection coverage to all wormhole types.

### 2.2. Packet Traversal Time (PTT) Measurement

As highlighted in Section 2.1, RTT is an inaccurate measure for determining the distance between two communicating nodes since packet processing delays on intermediate nodes have high variations. Conversely, the propagation speed of a data packet is nearly constant making the air traversal time of a packet much more accurate for distance estimations. The wormhole detection algorithm in TTHCA is thus based on the analysis of PTT rather than RTT.

The PTT between a source and a destination node is calculated during the AODV route discovery process by firstly measuring the RTT and then subtracting all packet processing delays incurred by intermediate nodes. A timer is started at the source node when a RREQ message is broadcasted and stopped when the corresponding RREP is received, giving the RTT. Each intermediate node receiving a RREQ measures *ΔT_RREQ_* and stores the measured value in its local memory. The destination node measures the time from receiving a RREQ until sending a RREP (*ΔT_RREQ_* + *ΔT_RREP_*) and adds this to the new parameter *ΔT_TOT_* in the AODV RREP packet. Upon receiving an RREP, all intermediate nodes measure *ΔT_RREP_* and increment *ΔT_TOT_* in the RREP packet by both *ΔT_RREP_* and the previously measured *ΔT_RREQ_* so: *ΔT_TOT_ = ΔT_TOT_ + ΔT_RREP_ + ΔT_RREQ_*. When the source node receives the RREP, it calculates the PTT for the route as:

(1) $$\mathrm{PTT}=\frac{\mathrm{RTT}-{\Delta T}_{\mathrm{TOT}}}{2}$$

In TTHCA, accurate PTT measurement must be provided by each intermediate node to enable the source node to effectively detect wormhole infected routes. In [18], IEEE 802.11b compliant hardware was shown to be able to measure data packet traversal times with an average error of between 3.9 ns and 7 ns depending on the network environment *i.e.*, indoors or outdoors. In Section 3.3, it will be proven TTHCA can tolerate much higher error measurement margins, resulting in no extra hardware being required to perform the time measurements.

### 2.3. TTHCA Wormhole Detection Algorithm and Extended AODV Route Discovery

While TTHCA defines a similar threshold to WAP for the maximum link delay per HC of a healthy route, it relaxes the fundamental assumption that network nodes have negligible packet processing delays by replacing RTT with PTT in the detection process, so a wormhole is suspected in a route if:

(2) $$\frac{\mathrm{PTT}}{\mathrm{HC}}>\frac{R}{S}$$

where *R* is the maximum radio range and *S* is the propagation speed of a wireless signal. This ratio sets the upper bound for all PTT per HC values, which means that no FP occurrences are feasible in TTHCA (see Section 3). Instead of performing the wormhole existence check on each intermediate node as in WAP, TTHCA uses a similar approach to that employed in DelPHI, to concentrate the check to the source node for minimising the route discovery delay.

**Figure 2.**
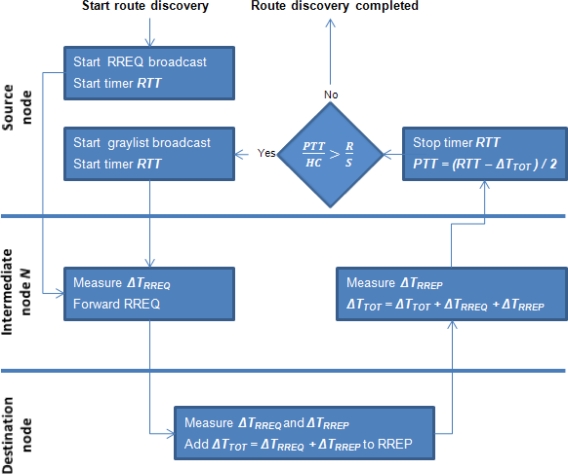
The TTHCA extended AODV route discovery algorithm.

The flowchart of the complete TTHCA extended AODV route discovery procedure is displayed in Figure 2.

During the AODV route discovery process *ΔT_TOT_* is measured and included in the RREP packet. When the source node receives a RREP packet, the PTT is calculated using Equation (1) and the threshold (2) applied to ascertain whether the identified route is potentially wormhole infected. If a wormhole is suspected, a graylist is broadcasted in an analogous manner to MHA to find a new unique route between the source and destination nodes. This iterative process of locating new routes is repeated until a safe route is found which unlike MHA, importantly makes TTHCA effective in HM wormhole detection. Furthermore, the network overhead is significantly reduced as only one route discovery iteration is needed if the MANET is wormhole free.

## 3. Results Analysis

The performance of TTHCA was rigorously tested in a MANET simulation environment developed in NS-2 [19], with all the simulation parameters being defined in Table 1.

**Figure 3.**
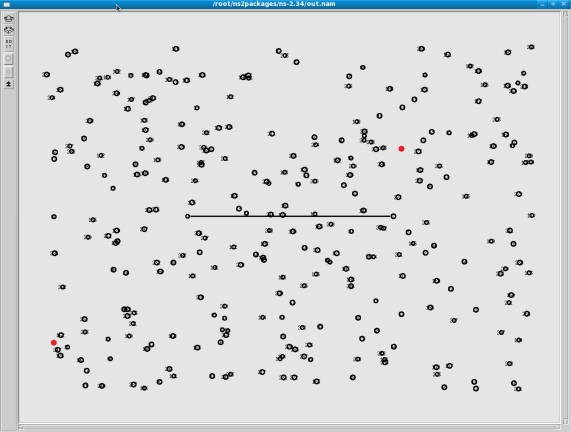
Sample environment from the NS-2 simulator showing a 4-hop PM O-B wormhole link, with the source and destination nodes in red.

It is assumed a single wormhole existed in the MANET with all nodes having identical IEEE 802.11b hardware and which, with the exception of the wormhole nodes, were randomly distributed within the network area. An example output from a NS-2 simulation run is displayed in Figure 3, where the source and destination nodes are shown in red and a 4-hop PM O-B wormhole link is located in the centre of the network.

MHA and DelPHI were recoded and respectively used as the comparative HC and RTT-based detection techniques for TTHCA in the simulation environment. The time threshold for DelPHI was *T* = 3 *ms* and *RREP_lim_* = 2 was chosen for MHA because in the case of only one wormhole, just two comparable route samples are sufficient for a wormhole route to be detected. Simulation results for WAP have not been included because this protocol assumes negligible packet processing delays on all intermediate nodes, which renders it unsuitable for this test environment which comprised nodes with realistic packet processing delays.

All four wormhole types with lengths between three and six hops were analysed, and the corresponding wormhole and FP detection rates for TTHCA, MHA and DelPHI calculated, with a statistical significance test performed to assess the confidence levels of the results. Wormhole lengths shorter than three hops were not included because they are not a significant network threat as they do not attract much traffic. Similarly, wormhole links longer than six hops inevitably produce a PTT/HC value which increases the likelihood of TTHCA successfully detecting the wormhole. To evaluate the performance limits of TTHCA, an analysis of the packet processing time measurement errors and radio range tolerances is also presented along with a route discovery delay overhead comparison between TTHCA, MHA and the original AODV protocol.

For each test case, simulation runs were repeated until 100 wormhole infected route samples were collected, so the total number of route samples was variable, (approximately in the range 300 to 1,100) and dependent both upon the network traffic attraction capability of the respective wormhole variant and the route discovery technique used by the respective wormhole detection scheme.

### 3.1. Wormhole Detection Performance and False Positive (FP) Occurrence

**Figure 4.**
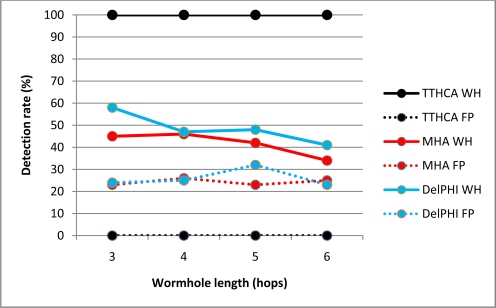
HM O-B wormhole (WH) detection and FP occurrence performance.

**Figure 5.**
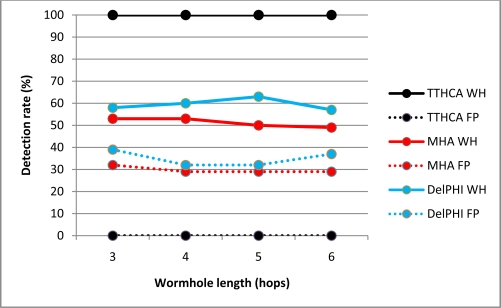
HM I-B wormhole (WH) detection and FP occurrence performance.

Figures 4 and 5 respectively show the detection performance for a HM I-B and O-B wormhole for various wormhole lengths, with the corresponding FP occurrences also plotted.

The detection results confirm that TTHCA outperformed both DelPHI and MHA for all wormhole lengths without generating any FP. HM wormholes are straightforward for TTHCA to detect because the malicious nodes do not modify the routing packets so their packet processing delays are not added to the RREP packets, causing the HM wormhole route to have a much higher PTT than a healthy route. This reflects the fact that the time to process a routing packet at an intermediate and/or wormhole node is much higher than for a packet to traverse two intermediate nodes. In the simulation, a notational packet processing delay of 4 ms was assumed, which compares with a typical PTT/HC value of ≈600 ns, so even very short HM and/or I-B wormhole links (1–2 hops) can be detected by TTHCA.

The lower detection and correspondingly higher FP rates for DelPHI are due to the high variations in routing packet processing delays. MHA displays a similar trend for both metrics, though in this case it reflects the fact that since nodes are randomly distributed in the network area, there are many instances where the wormhole link does not have the shortest HC, which decreases the detection rate and increases FP occurrences. Figures 4 and 5 also reveal that both MHA and DelPHI perform better for I-B link wormholes. For DelPHI, this is because an I-B wormhole has a higher link delay than an O-B wormhole, so an infected route is easier to distinguish from a healthy route. Due to its high link delay, an I-B wormhole does not attract as much traffic as an O-B wormhole, which lowers the instances of identifying a wormhole route not having the shortest HC, which tends to favour MHA.

**Figure 6.**
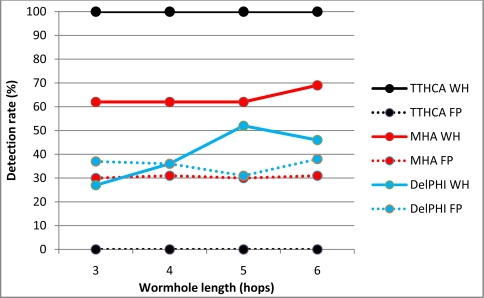
PM I-B wormhole (WH) detection and FP occurrence performance.

**Figure 7.**
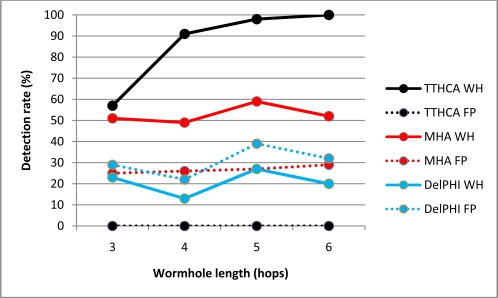
PM O-B wormhole (WH) detection and FP occurrence performance.

The equivalent results for a PM I-B and O-B wormhole are shown in Figures 6 and 7 respectively.

TTHCA provides superior wormhole detection for all I-B channel wormhole lengths. Despite PM wormhole nodes adding their packet processing delays to the RREP packets, the wormhole link has a high PTT because packets between the malicious nodes are tunnelled via legitimate nodes which do not add their respective packet processing delays to the RREP packets. Conversely, for an O-B wormhole link, TTHCA provided better than 90% detection accuracy for wormhole lengths of 4 or more hops. For shorter wormholes lengths however *i.e.*, three hops, the detection rate fell to below 60% due to the O-B wormhole having short link delays which makes the wormhole routes particularly difficult to discern from a healthy route. Overall, the TTHCA wormhole detection performance was still superior to both MHA and DelPHi, with interestingly no FP occurrences identified for all wormhole lengths analysed. As emphasised in Section 2.3, provided accurate packet processing time measurement is maintained then FP occurrences in TTHCA are not feasible, though as will be evinced in Section 3.3, when the measurement accuracy is relaxed, the corresponding FP occurrence rate commensurately increases.

The reason for the inferior detection performance of DelPHI for PM compared to HM wormholes is that a PM link is always two hops shorter than a HM link, so it incurs a shorter link delay. MHA in contrast, affords weaker HM detection performance because there are many instances where all routes located during the route discovery process traverse the wormhole, since as discussed in Section 1.1, HM wormhole nodes are not included in the graylist.

### 3.2. Statistical Significance Analysis

To quantitatively assess the comparative performance improvement of the TTHCA algorithm, a chi-square test of independence was undertaken to verify whether the wormhole detection rates and FP results are statistically significant compared with MHA and DelPHI. The detailed chi-square test results for all four wormhole variants are presented in Appendix, with a summary provided in Table 2, where the hypotheses are defined as follows:

$$\begin{array}{l} H_{0}:\text{TTHCA performance}\;=\;\text{MHA}/\text{DelPHI performance}\\ H_{1}:\text{TTHCA peroformance}\;\neq \;\text{MHA}/\text{DelPHI performance} \end{array}$$

The results conclusively prove that the difference in wormhole detection rates observed between TTHCA and DelPHI is statistically significant for all wormhole variants. Similarly for the TTHCA and MHA case, all observed wormhole detection performance differences are also significant except for the 3-hop PM O-B wormhole, where the null hypothesis was upheld for the reasons discussed earlier (see Figure 7). In terms of the FP occurrence performance, the null hypothesis is rejected in all cases.

### 3.3. Tolerance of Time Measurement Errors and Radio Range Variations in TTHCA

The next series of experiments analysed both the accuracy of packet processing time measurement and the maximum radio coverage variance per node necessary to maintain a predefined TTHCA wormhole detection rate of at least 75%. In a real-world MANET, the PTT measurement accuracy can deteriorate for a variety of reasons such as node mobility and limitations in the measurement capability of nodes during the route discovery process. Concomitantly, radio coverage can vary due to different MANET node hardware and variability in the network surroundings. These experiments were specifically performed on a PM O-B wormhole because this type of threat is the most challenging for TTHCA to detect.

**Figure 8.**
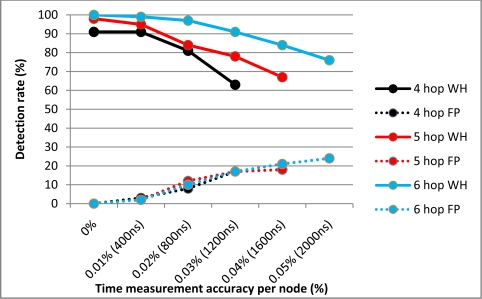
TTHCA time measurement error tolerance per node for a PM O-B wormhole (WH).

The time measurement error tolerance results are presented in Figure 8. A random error ξ = ±*K*% was introduced to each node measurement and the equivalent wormhole detection and FP rates analysed. *K* was set as a percentage of the median packet processing delay upon a MANET node (4 ms). The results confirm that relaxing the time measurement accuracy produced a correspondingly lower wormhole detection rate and higher FP incidence, since the measurements must be sufficiently accurate if they are to be compared with the median packet processing delay. Despite this constraint, the experiments revealed TTHCA was able to tolerate measurement errors up to ξ = 800 ns for all wormhole lengths, while still maintaining a detection rate of greater than 80%. This vindicates the premise in Section 2.2 that achieving satisfactory packet processing delay measurement accuracy on intermediate nodes is feasible without recourse for additional hardware.

**Figure 9.**
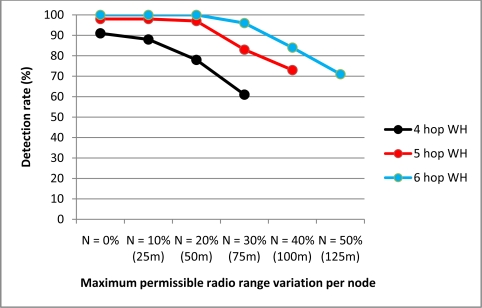
Radio coverage variation tolerances for TTHCA in detecting a PM O-B wormhole (WH).

The radio range variation results are displayed in Figure 9. A random variable γ representing the radio range loss per node is introduced, where 0 ≤ γ ≤ N% and N is the maximum permissible radio range variation per node, given the peak MANET node coverage is 250 m (see Table 1). Figure 9 reveals that higher variations in node radio coverage lead to a corresponding degradation in wormhole detection rates, though crucially TTHCA is still able to maintain a detection rate for wormholes of 4 or more hops of at least 75% when *N* = 20%, which corresponds to a radio range between 200 m and 250 m. Similarly for a 6-hop wormhole, the detection rate is at least 80% even for a MANET environment where *N* = 40% *i.e.*, a nodal radio range between 150 m and 250 m. Interestingly, variations in the radio range of MANET nodes did not lead to FP occurrences because the threshold employed by the TTHCA algorithm in (2) is based upon the maximum radio range per node, so radio coverage losses simply decrease the PTT/HC ratio for these routes.

### 3.4. Route Discovery Delay

Finally, to appraise the computational time overhead introduced by the TTHCA algorithm, the route discovery delay was calculated and compared with the original AODV protocol. MHA was also used in these experiments to demonstrate the reduced delay achieved by TTHCA in replacing *RREP_lim_* with the threshold in (2). Route discovery delays were measured for both a wormhole infected MANET environment, where a 4-hop PM O-B wormhole was assumed, together with a wormhole-free network. The results in Table 3 are based on the average route discovery delay for 100 route discovery samples for each protocol. They show that as TTHCA required only one route sample to identify a wormhole infected route, no delay in the route discovery procedure was introduced compared with AODV in a wormhole-free network. If a wormhole existed, a graylist broadcasting delay was incurred after a wormhole route had been identified so as to locate a new healthy route. MHA in contrast, nearly doubled its route discovery time compared to AODV, regardless of whether a wormhole existed or not. This is because it mandates that at least two unique routes must be identified to detect a wormhole. Note, these results do not take account of either the practical device delays caused by the packet processing time measurements or the increased RREP packet length, though pragmatically both of these represent a very small overhead for both the TTHCA and MHA algorithms.

## 4. Conclusions and Future Research

Wormhole attacks are one of the most severe threats to MANET routing and are difficult to detect as they can be launched in several modes, with each enforcing its own distinct requirements on the detection mechanism. This paper has presented a new robust wormhole detection algorithm based on Traversal Time and Hop Count Analysis (TTHCA) for the *Ad hoc* On-Demand Distance Vector (AODV) routing protocol. Simulation results, including a statistical significance test have proven TTHCA consistently provides superior wormhole detection performance allied with low false positive rates for all known wormhole types in a range of MANET scenarios, without incurring either significant computational or network cost. While the detection process is dependent on accurate packet processing time measurements on intermediate nodes, TTHCA has proven to be able to tolerate measurement errors well within acceptable bounds of existing wireless hardware.

In terms of future research, one important aim for the TTHCA algorithm is to ensure the integrity and accuracy of the packet processing time measurement (*ΔT*) on MANET devices and the prevention of possible measurement tampering. It is feasible that a participation mode wormhole node will deliberately give false measurement information concerning *ΔT*, so potentially compromising the wormhole detection mechanism. Research is focused upon evaluating the impact of malicious node measurement tampering for each wormhole type and investigating how TTHCA can be refined to ensure secure measurement and delivery of *ΔT*. One strategy being explored is replacing *ΔT_TOT_* in the route reply packet with a *ΔT* vector which includes the individual *ΔT* values from intermediate node, to afford the possibility of identifying suspicious *ΔT* measurements.

## Figures and Tables

**Table 1.** Simulation parameter settings.

**Parameter**	**Settings**

Number of nodes	300
Network area (*A*)	4,000,000 m^2^
Network width (*W*)	Random: 1,500 m–4,000 m
Network length (*L*)	*A/W*
Node wireless hardware	IEEE 802.11b
Maximum radio range / node (*R*)	250 m
Packet propagation speed (*S*)	300,000,000 m/s
Routing packet processing delay/node (*Average, Variance, Median*)	22 ms, 3 ms, 4 ms
Number of samples per test case:	
Wormhole infected routes (*r_wh_*)	100
Total number of routes (*r_tot_*)	*r_wh_* + all collected healthy routes
Output:	
Wormhole detection rate (%)	Number of identified wormhole routes / *r_wh_*
FP occurrence rate (%)	Falsely identified wormhole routes / *r_tot_*

**Table 2.** Statistical significance test results for wormhole detection and FP performance.

Wormhole variants		Result type	TTHCA *vs.* MHA	TTHCA *vs.* DelPHI
Type	Length (hops)			
HM I-B HM O-B PM I-B	3–6	Wormhole	*H_0_* = False	*H_0_* = False
		FP	*H_0_* = False	*H_0_* = False
PM O-B	3	Wormhole	*H_0_* = True	*H_0_* = False
		FP	*H_0_* = False	*H_0_* = False
	4–6	Wormhole	*H_0_* = False	*H_0_* = False
		FP	*H_0_* = False	*H_0_* = False

**Table 3.** Route discovery delay analysis for MHA and TTHCA compared with AODV.

**Protocol**	**Average delay**	
	**No WH**	**WH infected**

MHA	92%	98%
TTHCA	0%	43%

## Appendix

### Statistical Significance Test Results

The complete results from the chi-square test of independence are presented in Tables A1 and A2. These results verify the statistical significance of the observed differences between TTHCA and MHA, and TTHCA and DelPHI in terms of both wormhole detection and FP occurrences.

**Table A1.** Chi-square test results for wormhole detection rate differences for TTHCA *vs.* MHA and TTHCA *vs.* DelPHI.

Wormhole variants		TTHCA *vs.* MHA	TTHCA *vs.* DelPHI
Type	Length (hops)		
HM I-B	3	*X^2^*(3, *N_1,2_* = 100) = 61.44, *p* < 0.001	*X^2^*(3, *N_1,2_* = 100) = 53.16, *p* < 0.001
	4	*X^2^*(3, *N_1,2_* = 100) = 61.44, *p* < 0.001	*X^2^*(3, *N_1,2_* = 100) = 50.00, *p* < 0.001
	5	*X^2^*(3, *N_1,2_* = 100) = 66.67, *p* < 0.001	*X^2^*(3, *N_1,2_* = 100) = 45.40, *p* < 0.001
	6	*X^2^*(3, *N_1,2_* = 100) = 68.46, *p* < 0.001	*X^2^*(3, *N_1,2_* = 100) = 54.78, *p* < 0.001

HM O-B	3	*X^2^*(3, *N_1,2_* = 100) = 61.44, *p* < 0.001	*X^2^*(3, *N_1,2_* = 100) = 53.16, *p* < 0.001
	4	*X^2^*(3, *N_1,2_* = 100) = 73.97, *p* < 0.001	*X^2^*(3, *N_1,2_* = 100) = 72.11, *p* < 0.001
	5	*X^2^*(3, *N_1,2_* = 100) = 81.69, *p* < 0.001	*X^2^*(3, *N_1,2_* = 100) = 70.27, *p* < 0.001
	6	*X^2^*(3, *N_1,2_* = 100) = 98.51, *p* < 0.001	*X^2^*(3, *N_1,2_* = 100) = 83.69, *p* < 0.001

PM I-B	3	*X^2^*(3, *N_1,2_* = 100) = 46.91, *p* < 0.001	*X^2^*(3, *N_1,2_* = 100) = 114.96, *p* < 0.001
	4	*X^2^*(3, *N_1,2_* = 100) = 46.91, *p* < 0.001	*X^2^*(3, *N_1,2_* = 100) = 94.12, *p* < 0.001
	5	*X^2^*(3, *N_1,2_* = 100) = 46.91, *p* < 0.001	*X^2^*(3, *N_1,2_* = 100) = 63.16, *p* < 0.001
	6	*X^2^*(3, *N_1,2_* = 100) = 36.69, *p* < 0.001	*X^2^*(3, *N_1,2_* = 100) = 73.97, *p* < 0.001

PM O-B	3	*X^2^*(3, *N_1,2_* = 100) = 0.72, *p* = 0.867	*X^2^*(3, *N_1,2_* = 100) = 24.08, *p* < 0.001
	4	*X^2^*(3, *N_1,2_* = 100) = 42.00, *p* < 0.001	*X^2^*(3, *N_1,2_* = 100) = 121.88, *p* < 0.001
	5	*X^2^*(3, *N_1,2_* = 100) = 45.06, *p* < 0.001	*X^2^*(3, *N_1,2_* = 100) = 107.54, *p* < 0.001
	6	*X^2^*(3, *N_1,2_* = 100) = 63.16, *p* < 0.001	*X^2^*(3, *N_1,2_* = 100) = 133.33, *p* < 0.001

**Table A2.** Chi-square test results for FP occurrence rate differences for TTHCA *vs.* MHA and TTHCA *vs.* DelPHI.

Wormhole variants		TTHCA *vs.* MHA	TTHCA *vs.* DelPHI
Type	Length (hops)		
HM I-B	3	*X^2^*(3, *N_1_* = 506, *N_2_* = 1,072) = 207.64, *p* < 0.001	*X^2^*(3, *N_1_* = 506, *N_2_* = 665) = 255.56, *p* < 0.001
	4	*X^2^* (3, *N_1_* = 510, *N_2_* = 953) = 179.61, *p* < 0.001	*X^2^*(3, *N_1_* = 510, *N_2_* = 627) = 201.01, *p* < 0.001
	5	*X^2^* (3, *N_1_* = 516, *N_2_* = 712) = 180.43, *p* < 0.001	*X^2^*(3, *N_1_* = 516, *N_2_* = 641) = 204.14, *p* < 0.001
	6	*X^2^* (3, *N_1_* = 520, *N_2_* = 735) = 178.43, *p* < 0.001	*X^2^*(3, *N_1_* = 520, *N_2_* = 708) = 248.19, *p* < 0.001

HM O-B	3	*X^2^* (3, *N_1_* = 291, *N_2_* = 359) = 78.19, *p* < 0.001	*X^2^*(3, *N_1_* = 291, *N_2_* = 271) = 78.93, *p* < 0.001
	4	*X^2^* (3, *N_1_* = 300, *N_2_* = 383) = 89.62, *p* < 0.001	*X^2^*(3, *N_1_* = 300, *N_2_* = 316) = 86.03, *p* < 0.001
	5	*X^2^* (3, *N_1_* = 302, *N_2_* = 431) = 81.14, *p* < 0.001	*X^2^*(3, *N_1_* = 302, *N_2_* = 323) = 116.65, *p* < 0.001
	6	*X^2^* (3, *N_1_* = 310, *N_2_* = 365) = 90.47, *p* < 0.001	*X^2^*(3, *N_1_* = 310, *N_2_* = 381) = 101.87, *p* < 0.001

PM I-B	3	*X^2^* (3, *N_1_* = 667, *N_2_* = 1,144) = 249.38, *p* < 0.001	*X^2^*(3, *N_1_* = 667, *N_2_* = 772) = 308.39, *p* < 0.001
	4	*X^2^* (3, *N_1_* = 690, *N_2_* = 1,027) = 260.19, *p* < 0.001	*X^2^*(3, *N_1_* = 690, *N_2_* = 750) = 307.12, *p* < 0.001
	5	*X^2^* (3, *N_1_* = 679, *N_2_* = 1,045) = 249.22, *p* < 0.001	*X^2^*(3, *N_1_* = 679, *N_2_* = 541) = 248.13, *p* < 0.001
	6	*X^2^* (3, *N_1_* = 685, *N_2_* = 1,059) = 259.10, *p* < 0.001	*X^2^*(3, *N_1_* = 685, *N_2_* = 678) = 318.97, *p* < 0.001

PM O-B	3	*X^2^* (3, *N_1_* = 468, *N_2_* = 539) = 133.05, *p* < 0.001	*X^2^*(3, *N_1_* = 468, *N_2_* = 321) = 151.84, *p* < 0.001
	4	*X^2^* (3, *N_1_* = 519, *N_2_* = 474) = 150.87, *p* < 0.001	*X^2^*(3, *N_1_* = 519, *N_2_* = 296) = 125.92, *p* < 0.001
	5	*X^2^* (3, *N_1_* = 491, *N_2_* = 563) = 154.90, *p* < 0.001	*X^2^*(3, *N_1_* = 491, *N_2_* = 378) = 231.71, *p* < 0.001
	6	*X^2^* (3, *N_1_* = 579, *N_2_* = 538) = 195.14, *p* < 0.001	*X^2^*(3, *N_1_* = 579, *N_2_* = 344) = 210.20, *p* < 0.001

The nomenclature used in these Tables is: *X^2^* is the chi-square value, *N_1_* is the number of route samples used as the basis for the TTHCA results; *N_2_* is the corresponding number of MHA/DelPHI route samples and *p* is the probability the observed difference between TTHCA and MHA/DelPHI is not statistically significant.
